# Earthquakes and extreme rainfall induce long term permeability enhancement of volcanic island hydrogeological systems

**DOI:** 10.1038/s41598-020-76954-x

**Published:** 2020-11-19

**Authors:** B. Vittecoq, J. Fortin, J. Maury, S. Violette

**Affiliations:** 1 BRGM, 97200 Fort-de-France, Martinique; 2 CNRS, UMR.8538 – Laboratoire de Géologie, ENS-PSL Research University, 24 rue Lhomond, 75231 Paris, France; 3 BRGM, 45060 Orléans, France; 4 Sorbonne University, UFR.918, 75005 Paris, France

**Keywords:** Hydrogeology, Seismology

## Abstract

Earthquakes affect near-surface permeability, however temporal permeability evolution quantification is challenging due to the scarcity of observations data. Using thirteen years of groundwater level observations, we highlight clear permeability variations induced by earthquakes in an aquifer and overlaying aquitard. Dynamic stresses, above a threshold value PGV > 0.5 cm s^−1^, were mostly responsible for these variations. We develop a new model using earth tides responses of water levels between earthquakes. We demonstrate a clear permeability increase of the hydrogeological system, with the permeability of the aquifer increasing 20-fold and that of the aquitard 300-fold over 12 years, induced by fracture creation or fracture unclogging. In addition, we demonstrate unprecedented observations of increase in permeability due to the effect of extreme tropical deluges of rainfall and hurricanes. The water pressure increase induced by the exceptional rainfall events thus act as piston strokes strong enough to unclog congested fractures by colloids, particles or precipitates. Lastly, an analysis of regional permeabilities also highlights a permeability increase over geological timeframes (× 40 per million years), corroborating the trend observed over the last decade. This demonstrates that permeability of aquifers of andesitic volcanic islands, such as the Lesser Antilles, significantly evolve with time due to seismic activity and extreme rainfall.

## Introduction

Among the many direct or indirect effects of earthquakes, various hydrogeological responses have been observed. The most striking are water level oscillations, water level co-seismic or post-seismic drop or rise, from centimetric to pluri-metric scale (e.g. Refs.^1–3^), new streams and springs appearance^4^, disappearance of existing springs, streams discharge increase. These impacts are observed from near field to hundreds to thousands of kilometers from the earthquake, depending on its magnitude^2,5^. In the near field, these modifications could directly affect water resource production, its quality, and supply to the population.

Such observations of interaction between seismic waves propagation and groundwater are precious opportunities to investigate the hydrogeological functioning of aquifers and their hydraulic properties evolution over time^5^, especially in confined aquifer where boreholes react as natural strain meters^6^. Earthquakes induce static and dynamic stress changes, as a function of distance and magnitude, and such changes can induce aquifer permeability changes^2,7–10^. However, relating field-scale observation to a mechanism is challenging as mechanisms occur mainly at the pore-scale, which cannot be easily observed on site.

The main mechanisms leading to temporary or permanent increase or decrease of aquifer permeability are due to: (a) pore pressure variation and fracture aperture variations induced by change in effective pressure—defined as mean stress minus pore pressure—^11–14^, (b) clogging/unclogging of fracture due to remobilization of deposits and particles^2,8,9,15,16^, (c) creation of new fractures^10,17,18^, but also (d) consolidation or liquefaction^19,20^ or (e) release of vadose zone^21,22^. An increase in permeability related to fracture unclogging, an often-mentioned mechanism in fractured media, is usually followed by a return to pre-earthquake permeability value over a period of months to years^2,9,17,23^.

A decrease in permeability triggered by earthquakes is much less documented^24–27^. In this last case, fractures clogging by particles or fine sediments is often involved, with a possible link with earthquake azimuth relative to fracture orientation^27^. Over time, permeability increase and decrease can be observed in the same boreholes^27^. Lastly, permeability variations not only concern aquifers but also aquitards^18,28^ whose hydraulic properties increase can have significant environmental consequences, an example is a leak off from polluted subsurface water to underground water.

Characterizing and quantifying temporal evolution of permeability, the key hydraulic parameter controlling groundwater flow, also remain one of the present-day scientific challenges^5,29^. Long term monitoring is difficult and challenging^30^ but monitoring permeability evolution in seismically active regions such as subduction zones could provide unique opportunities to understand the link between earthquakes and permeability variations.

**Figure 1:**
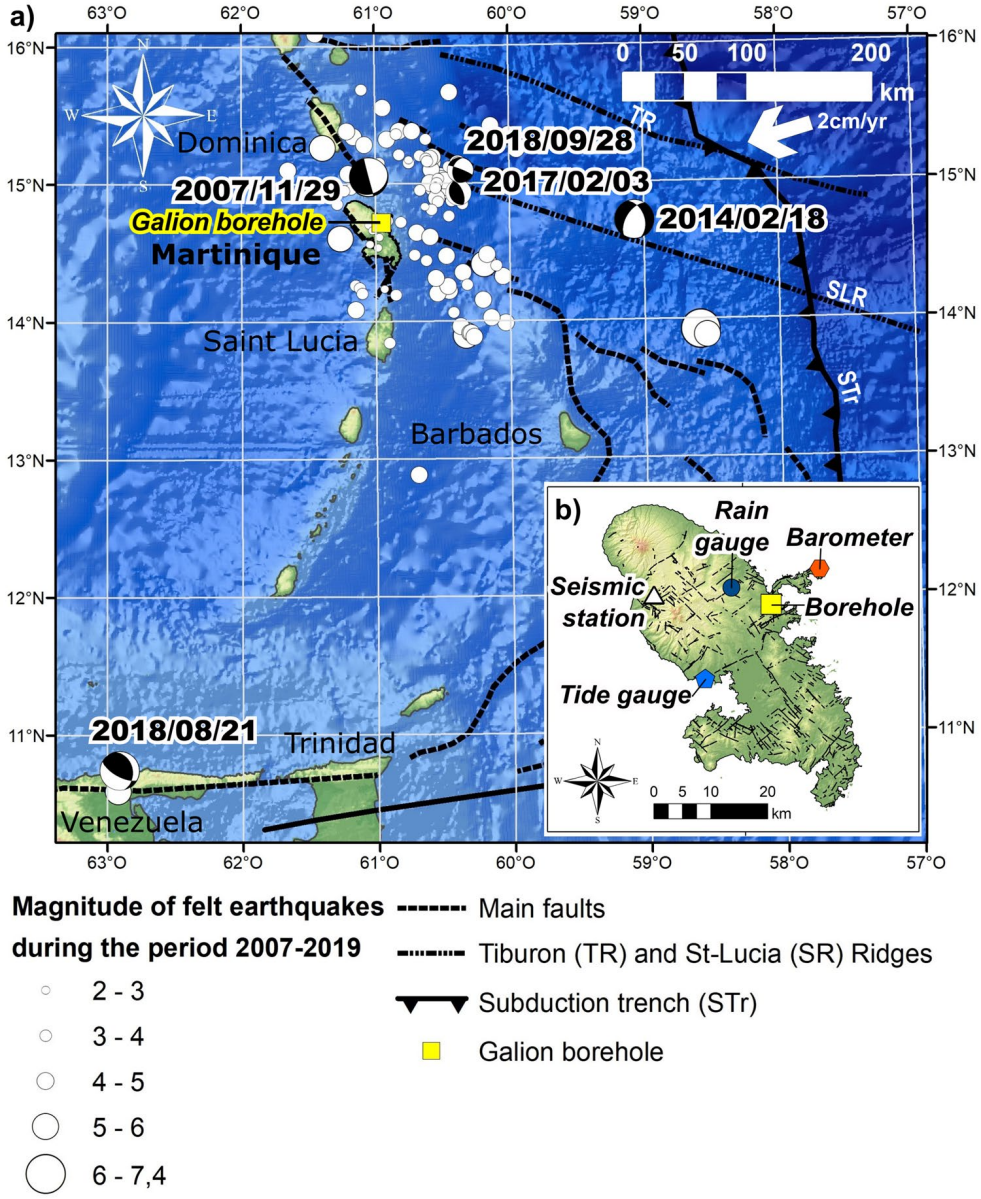
(**a**) Localization of the 128 felt earthquakes in Martinique reported by the OVSM-IPGP observatory during the period 2007–2019. Moment tensors of the five strongest earthquakes: M7.4—2007/11/29 (hypocentral distance—hd: 163 km), M6.5—2014/02/18 (hd: 203 km), M5.6—2017/02/03 (hd: 74 km) M7.3 2018/08/21 (hd: 485 km) and M5.4 2018/09/28 (hd: 90 km). Digital elevation model from the General Bathymetric Chart of the Ocean (gebco.net), vector of convergence^32^, main faults, ridges and subduction trench^69^. (**b**) Localization of the Galion Borehole, the ocean tide gauge (Fort-de-France harbor), the barometer station (barometric pressure data), the rain gauge station, and the seismic station of the volcanic and seismologic observatory of Martinique (OVSM-IPGP). Figure performed using ArcGis 10.5.1 (https://www.esri.com/).

**Figure 2:**
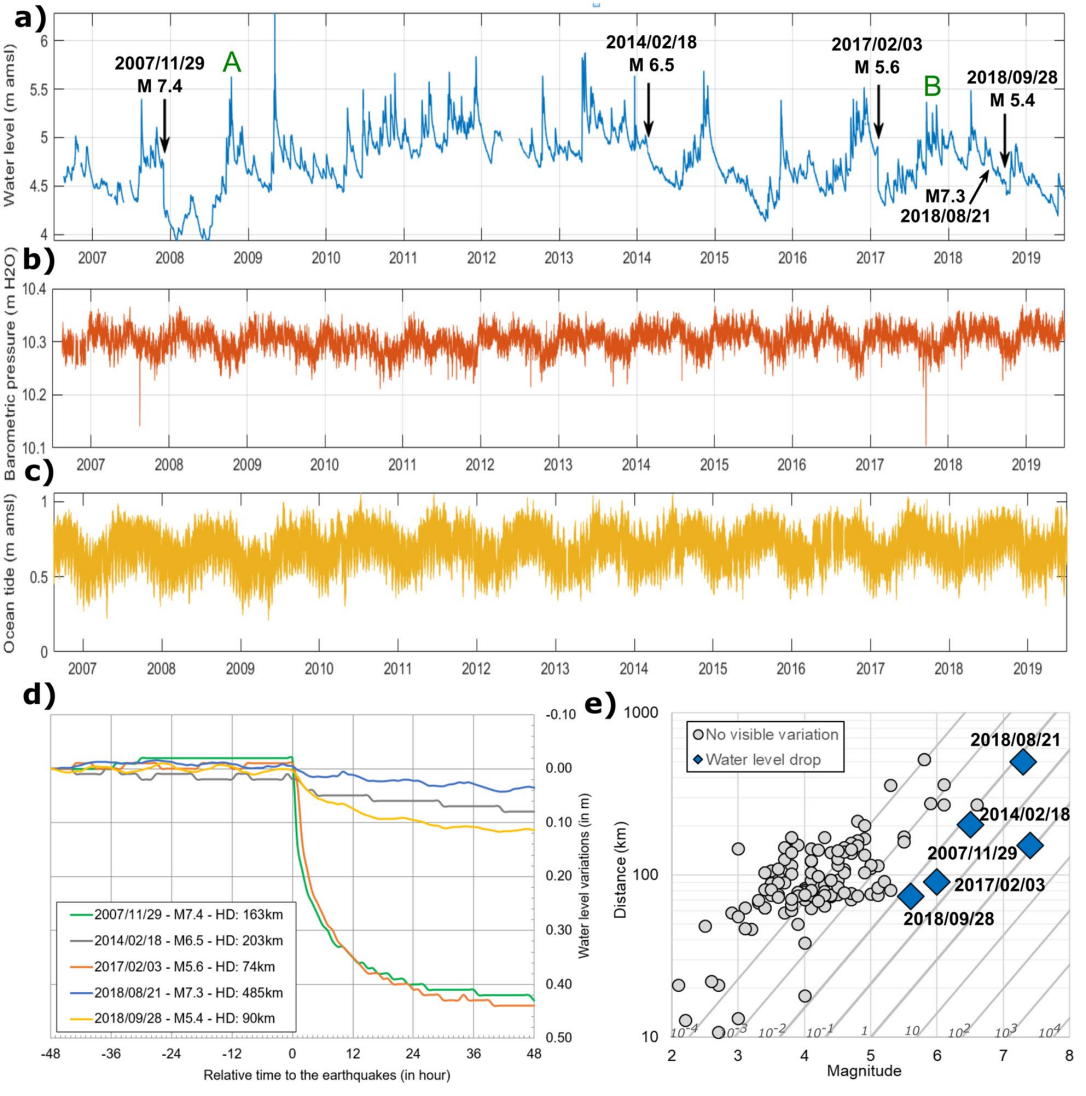
The five most important earthquakes, with a seismic energy density higher than 1.6 × 10^–2^ J m^−3^, have triggered co-seismic water-levels drop from 2 to 44 cm in the Galion borehole. The borehole is 50 m deep and intersects fissured and fractured 15 Ma years’ old basalts between 19 and 50 m (screened interval), with a 19 m thick layer of very low pervious clay recovering (called aquitard). The aquifer, confined with a + 4.5 m water level, above mean sea level (amsl), flows toward the sea located 1.5 km east. Time series data from August 2006 to December 2019, at a one-hour sampling rate, of (**a**) groundwater level in Galion borehole (in m above mean sea level − amsl) (A = October 2008 extreme rainfall and B = cat.5 Irma and Maria Hurricanes), (**b**) barometric pressure at the nearest meteorological station (10 km NE) and (**c**) Ocean tide at Fort-de-France harbor. (**d**) Relative water level variations following the five strongest earthquakes compared to a fixed “0 m” level set 48 h before. (**e**) Distribution of earthquakes as a function of magnitude and hypocentral distance. Oblique lines are the seismic energy density in J m^−3^ ^37^.

The Lesser Antilles slow-spreading subduction zone^31^ is characterized by a relatively old subducting plate (80 Ma), with a subduction rate of 19 mm/year^32,33^. Martinique Island is the largest volcanic island (1080 km^2^) of the Lesser Antilles archipelago, with a volcanic activity, mainly andesitic, for at least 25 Ma^34^. Climate in Martinique is tropical, marked by a dry season and a rainy season, interspersed with fluctuating transition periods. The average frequency of cyclones in the north Atlantic is around 12 per year, and the maximum daily rainfall recorded in the Galion watershed is 480 mm in a day (Hurricane Klaus, October 4, 1990). Historically thirty destructive earthquakes (intensity > VI) have impacted Martinique Island between 1702 and 2019, including 6 with intensity between VII and VIII and one major in 1839 with an intensity of IX^35^. Over the period 2007–2019, 130 earthquakes were felt in Martinique (Fig. 1) and reported by the Volcanological and Seismological Observatory of Martinique (OVSM-IPGP) (min magnitude 2.1, median 4.2 and max 7.4). The earthquakes occurring around Martinique Island are subduction earthquakes and shallower events showing shortening and overthrusting^36^. Shallow events from north of Martinique show more normal and strike-slip events expressing trench-parallel extension. The five most important earthquakes felt in Martinique in the past thirteen years (2007/11/29, 2014/02/18, 2017/02/03, /2018/08/21 and 2018/09/28) have triggered co-seismic water-levels drop from 2 to 44 cm in the Galion borehole, located on the east coast of the island (Figs. 1, 2a,d), monitored at an hourly time step since December 2005. These earthquakes are varied in term of depth (from 15 to 160 km), azimuth relative to the Galion borehole (Fig. 1), distance (from 38 to 485 km) and mechanism (thrust or normal events, with some strike-slip component). Furthermore, their seismic energy density is higher than 1.6 × 10^–2^ J m^−3^ (Fig. 2e) following an empirical function^37^, that estimates seismic energy density as a function of hypocentral distance and earthquake magnitude.

In this article, we take advantage of this unique long piezometric monitoring in the Lesser Antilles to report unprecedented observations of permeability variations induced by earthquakes. In addition, we highlight the superimposed effect of exceptional tropical rain and hurricanes on the permeability increase. Our results combined with a compilation of transmissivities calculated by pumping tests performed in boreholes spatially distributed at the scale of the Martinique Island also reveal that permeability of aquifers of this andesitic volcanic island sustainably increases with age due to these two processes: seismic activity and extreme rainfalls.

## Results

### Transmissivity increase of the hydrogeological system (aquifer and aquitard) over 12 years and over geological ages

**Figure 3:**
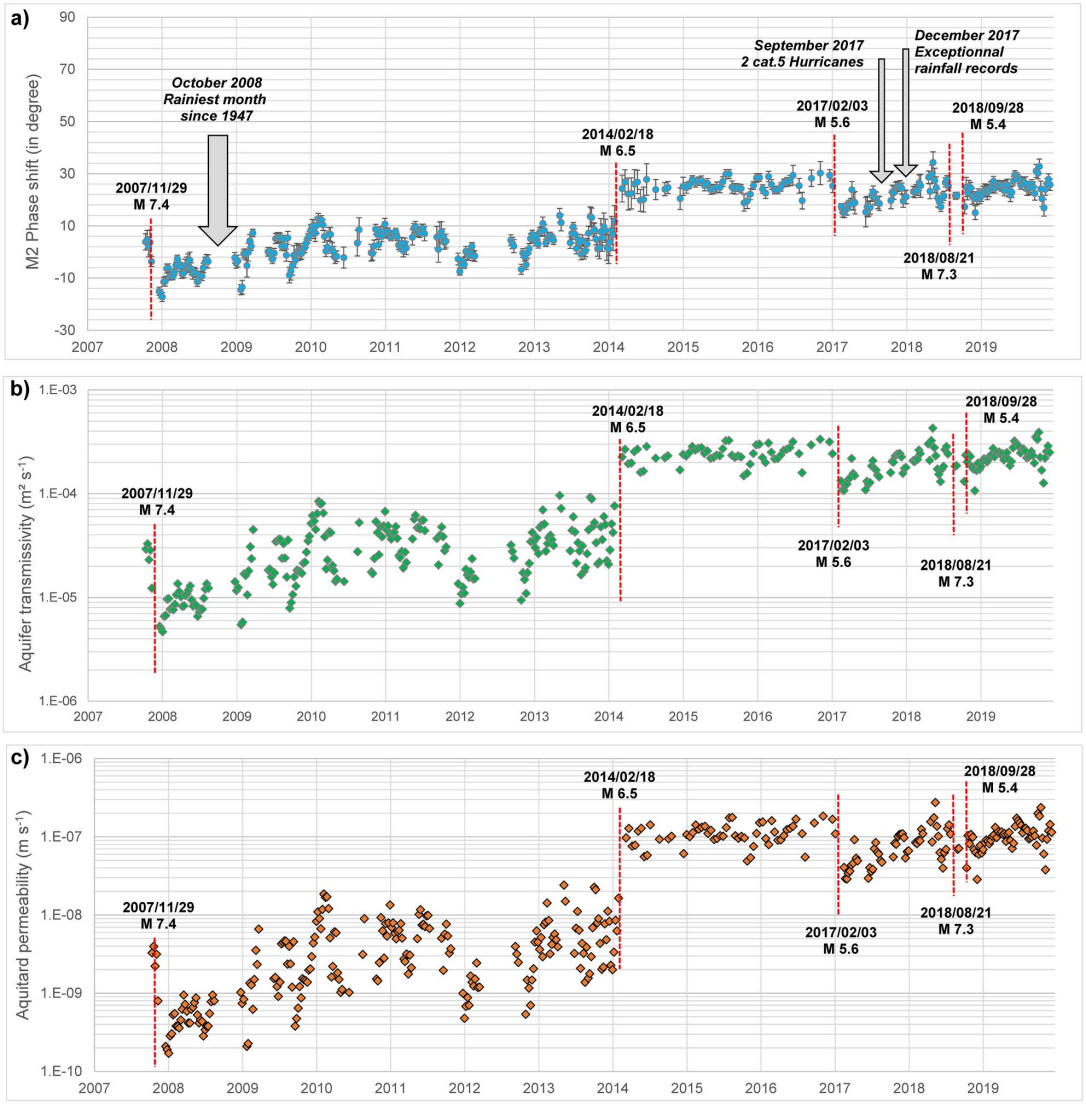
M2 phase shift and permeability evolution of the hydrogeological system over 12 years. (**a**) Phase shift (Phase shift error bars correspond to ± the root-mean-square error—RMSE) between Galion borehole water levels and M2 earth-tide calculated with Baytap08^40^, (**b**) Aquifer transmissivity and (**c**) Aquitard permeability, both calculated with our analytical model.

Aquifer transmissivity can be determinated from earth tide analysis^9,17,27,28,38^, considering the time lag (phase shift) between the tidal dilatation of the aquifer and the water level response in the well^6^. Geologic or topographic heterogeneity may affect the tidal response of an area^39^, but these effects did not vary over the duration of our study^10^, and should not affect the phase lag evolution. We use Baytap08 software^40^ to calculate, by period of 31 days with an overlay of 15 days, the phase shift of the water-level response to M2 tidal component of earth tides (Fig. 3a). Significant changes in the phase of the tidal responses are observed following the five earthquakes, with values ranging from − 17° to  + 34°. The 2014/02/18 earthquake induces a clear increase of the tidal phase shift (+ 21°), whereas 2007/29/11 and 2017/02/03 earthquakes induce a decrease of the tidal response (− 19° and − 9°, respectively).

Several models have been developed and tested to calculate transmissivity from tidal response of water level. They take into account different hypotheses and can therefore be applied only in certain cases: confined isotropic aquifer, radial flow and negative phase shift^6^, unconfined aquifer, vertical flow and positive phase shift^41,42^. Recently a new model considering both horizontal flow and vertical leakage allows to consider both negative and positive phase shift^43^, and put in evidence that the phase shift sign is not a trustworthy condition for defining if an aquifer is confined or unconfined. In our case, we have to consider a confined aquifer, with horizontal flow, possible leakage from overlying aquitard, and phase shift both positive and negative. We choose to improve this last model^43^ in which the phase shift for a given period (O1, M2, N2) is function of the storativity *S* and transmissivity *T* of the aquifer and the ratio permeability versus thickness of the aquitard *k/b.*

**Figure 4:**
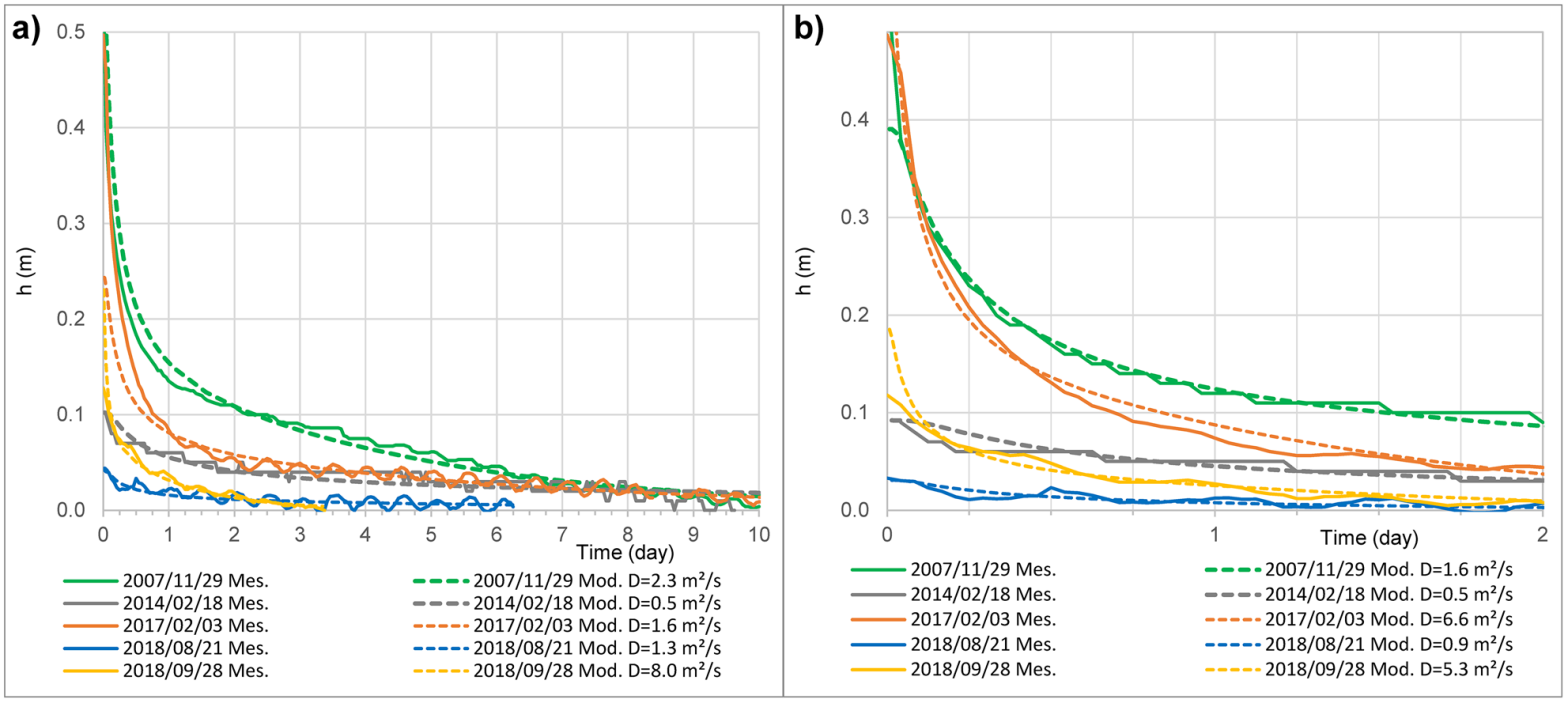
Co-seismic drawdown modelling. (**a**) Water level drop during the 10th first days, and (**b**) refinement on the 48th first hours after earthquakes, have been modelled considering a simple one-dimensional model of confined aquifer^44^. Results show a low diffusivity variability (mean = 3 m^2^ s^−1^, standard deviation = 3 m^2^ s^−1^). These water levels drop match with type 3 (down-down) responses^44^, as the groundwater level continued to decline following a co-seismic fall, associated with a sudden increase in transmissivity of the aquifer.

To constrain the model in term of input, water levels drop during the 10 days after the five strongest earthquakes have been modeled (Fig. 4) considering a one-dimensional model of confined aquifer^44^. This model allows us to characterize the diffusivity (D = T/S) of aquifer just after each earthquake. These water level drop match with type 3 (down-down) responses classification^44^, as the groundwater level continued to decline following a coseismic fall. Type 3 responses are associated with a sudden increase in the porosity, which could be caused by unclogging or creation of new fractures, inducing a sudden decrease in the water level^44^. Results show a relatively low diffusivity variability, ranging from 0.5 to 8 m^2^ s^−1^ with a mean value of 3 m^2^ s^−1^ and a standard deviation of 3 m^2^ s^−1^ (Fig. 4). This low variability in diffusivity suggests that transmissivity and storage coefficient varied jointly.

We develop an analytical model improving the aquifer model with an overlying aquitard^43^ by adding two assumptions: (1) diffusivity T/S is constant and (2) the permeability of the aquifer varies exponentially with stress^45,46^. Then, we deduce a relationship between variation in permeability in the aquifer and variation in permeability in the aquitard. Finally, in this improved model, we are able to express the phase shift for a given period as a function of only one independent parameter: the transmissivity of the aquifer. This new model is calibrated with two transmissivity values (one in 2019 calculated from a pumping test and the other in 2008 calculated from the phase shift values of O1 and M2 earth tide waves). This model thus makes it possible to deduced the evolution of the permeability over the 12 years (Fig. 3b,c) from the temporal evolution of the M2 tidal phase shifts. Note that the permeability inferred from tidal analysis is always a mean permeability estimate over a given period (31 days in our case). Results put in evidence transmissivity increase from 9 × 10^–6^ and 3 × 10^–4^ m^2^ s^−1^ for the aquifer and permeability increase between 4 × 10^–10^ and 1 × 10^–7^ m s^−1^ for the aquitard. We thus demonstrate a clear permeability increase of the hydrogeological system, with a permeability of the aquifer multiplied by 20 and that of the aquitard by 300 in 12 years.

**Figure 5:**
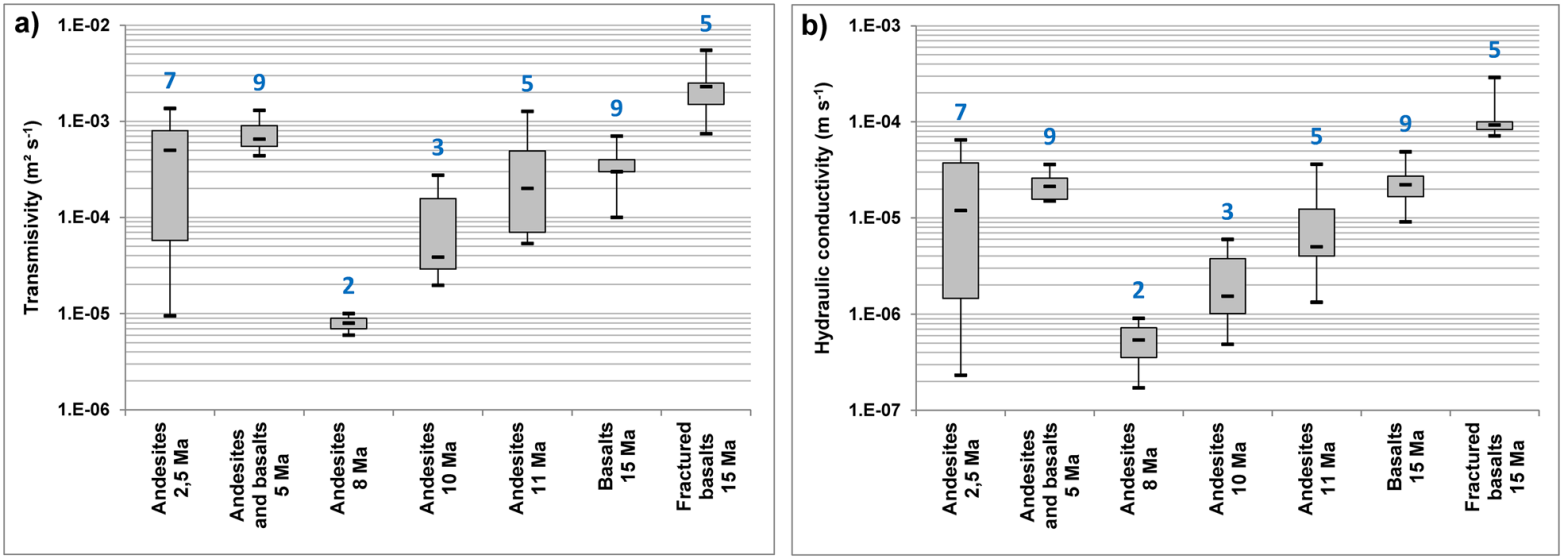
Aquifer transmissivity and hydraulic conductivity increase over geological ages. Synthesis of: (**a**) Transmissivity and (**b**) Hydraulic conductivity data calculated from pumping tests in 40 boreholes, drilled in last decades in fissured and fractured volcanic aquifers in Martinique, representing seven different geological formations with age ranging from 2.5 to 15 Ma. Box boundaries are first and third quartile; black dashes correspond to min, max and median. The blue numbers correspond to the number of boreholes in each category.

In order to assess whether the trends observed over 12 years could be projected over a longer period, we collect permeability data calculated from pumping tests in 40 boreholes, drilled in last decades in fissured and fractured volcanic aquifers in Martinique, representing seven different geological formations with age ranging from 2.5 Ma to 15 Ma (Fig. 5). Geological formations with age between 2.5 and 5 Ma have been set up during aerial volcanism phases and those between 8 and 15 Ma during older submarine volcanism phases. For the two different phases, we highlight a permeability increases with age over geological ages. Between 8 and 15 Ma, permeability increased by a factor of 300 (ie. × 40 per million years).

### Permeability increase following post-seismic intense rainfall and hurricane events

Following 2007/11/29 and 2017/02/03 earthquakes, the tidal analysis shows for the two following months a permeability decrease of the aquifer (Fig. 3), respectively from 2.9 × 10^–5^ m^2^ s^−1^ to 5.3 × 10^–6^ m^2^ s^−1^ and from 2.4 × 10^–4^ m^2^ s^−1^ to 1.1 × 10^–4^ m^2^ s^−1^. However, we can observe, in the following few months, a gradual permeability increase, and, in both cases, a return to pre-earthquake value over a period of months to years (with no earthquakes with significant seismic energy during these periods). Interestingly, several major meteorological events occurred in the next months following these earthquakes (Fig. 3a). October 2008 is the rainiest month since 1947 with 500–700 mm in the borehole watershed (Mean October pluviometry: 300 mm). 2017 is also exceptional, with two major’s hurricanes (Cat. 5 Irma and Maria) crossing the Lesser Antilles arc in September 2017. This month becomes one of the wettest months since decades, with 400–450 mm in the watershed (Mean pluviometry in September: 270 mm). Daily record is also exceptional with more than 200 mm in a day when Maria hurricane was closer to the island (2017/09/18). Finally, December 2017 also show exceptional rainfall records (400–500 mm) with more than 110 mm in a day (Mean pluviometry in December: 210 mm).

**Figure 6:**
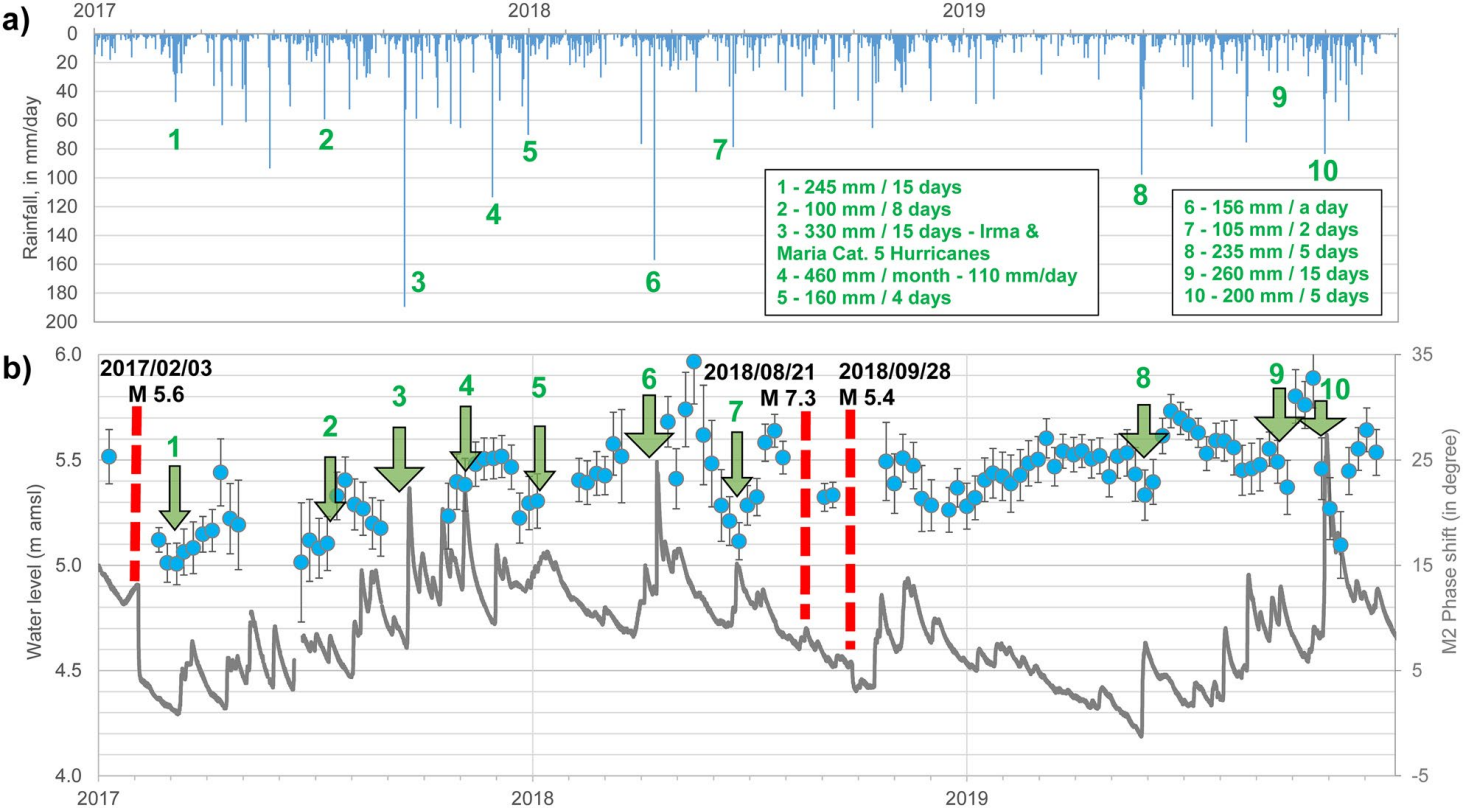
Phase shift increase following major meteorological events. (**a**) Daily rainfall from 2017 to 2019. (**b**) Galion borehole water level fluctuations versus M2 phase shift. Comparing water level evolution and M2 phase shift variations from 2017 to 2019 put in evidence M2 phase shift increases following major meteorological events and following the 2017/02/03 earthquake. These meteorological events act as piston strokes strong enough to unclog congested fractures by colloids, particles or precipitates, further confirming that the mechanism decreasing permeability occurring following the 2017/02/03 earthquake could be fracture clogging. (The phase shift error bars correspond to ± the root-mean-square error -RMSE).

Significant rainfalls have also been recorded during 2018 and 2019. Figure 6 shows the evolution of rainfalls, water level fluctuations and M2 phase shift variations from 2017 to 2019. Groundwater level fluctuations show two trends. First, a seasonal trend, with a global trend of water level increase during rainy season and a global trend of water level decrease during dry season. Superimposed on this first trend, a short-term pluviometric trend is observed, with fast water level increase (few hours) following intense rainfalls. This latter trend, directly and quickly impacted by rainfall, is correlated with M2 phase shift increase following the ten events identified in Fig. 6. We can also observe, for some events, permeability decrease following intense rainfalls permeability increase (e.g. following events 2, 6 or 8 on Fig. 6) suggesting particles or colloids movements and reclogging, but the general trend, over three years, is a permeability increase. These results thus highlight that major meteorological events have an impact on the aquifer permeability increase, as these events, acting as pistons strokes, induce fracture unclogging process.

### Effect of static and dynamic stresses and strains

Both static and dynamic stresses can cause aquifer permeability changes^2,8,9^. In the near field (i.e. distance equivalent to the length of the rupture fault), static and dynamic strains are comparable in magnitude^37^. In the far field, at greater distances, more than ten times the rupture length^47^, dynamic stresses (that decrease in 1/r^2^ to 1/r^3/2^ depending on the wave) are much greater than static stresses (that decrease in 1/r^3^). Based purely on distance criterion, the Galion borehole is located in the intermediate to far field of the five most important earthquakes. Therefore, it is not clear whether the water level drop observed can be linked to a static stress change or a dynamic stress change. With a long-term impact, it would be expected that static stress change modified the aquifer properties^13^ but some researchers suggest that dynamic stress change can also have long-term consequences even if the mechanism involved is not clear^2,8,12^.

We calculate the static mean stress change with the Okada Model^48^ for the eight most important earthquakes over the period 2007–2019 (SI Appendix, Table S1), among which five triggered a permeability change. Results ranges from a few Pascal to tenth Pascal, except for 2007 and 2014 earthquakes with a variation of around 1.6 kPa and 0.3 kPa respectively (SI Appendix, Fig. S1). We also calculate the change in pore pressure (SI Appendix, Table S1) assuming a Skempton coefficient B between 0.6 and 0.9^41–49^. The 2007 and 2014 earthquakes induce a change in water level of 10–15 cm and 2–3 cm, respectively. For these two events, an impact of static stress change on the water level is possible, as observed variations at the Galion piezometer are of the same order, 43 cm and 8 cm, respectively (Fig. 2a,d).

Static effect changes are yet not able to explain groundwater changes observed for the others earthquakes. For 2017 and 2018 earthquakes, the static mean stress variations estimated are too low (0.01 to 0.1 kPa) compared to earth tide influence (kPa)^50^. Furthermore, if static stress changes were responsible, higher water level change would be associated to the higher static stress change, which is not the case for 2017/02/03 earthquake with the higher water level change (0.44 cm) and low static stress (25 Pa).

The impact of dynamic stresses can be estimated through the peak ground velocity (PGV) of a seismic signal, assuming plane wave propagation, which can be linked to the peak dynamic shear stress (e.g.^51–53^). We then calculate PGV (maximum value of the 3D vector) for the eight most important felt earthquakes with data from the seismic station located at the OVSM-IPGP, 20 km from the Galion piezometer, as it is the only station on the island that registered all earthquakes considered (SI Appendix, Table S1). Results show that earthquakes that occurred just before a water level change of more than 2 cm have PGV higher than 0.5 cm s^−1^. Interestingly the 2007 and 2017 earthquakes have the higher PGV values (2.1–2.8 cm s^−1^) and they are also the earthquakes that triggered the higher changes in water level (0.4 m) (SI Appendix, Fig. S2). Dynamic stresses are then clearly correlated to -and responsible of- the observed co-seismic water level drop.

## Discussion

This article is the first published study characterizing the evolution of the permeability of a volcanic island hydrogeological system (aquifer and aquitard) due to the impact of the seismic activity of a subduction zone with cumulative effect of major tropical rainfalls. The permeability increase highlighted over the last twelve years is corroborated over geological ages thanks to the statistical analysis of existing pumping tests data in comparable aquifers of the island. Since we highlight over twelve years that permeability globally increase over the period, but that some earthquakes can also decrease permeability, it follows that permeability decrease should be transient because of: i) the tropical climate with hurricane and extreme rainfall regularly flushing particles and thus unclogging fractures, ii) the possible effect of successive earthquakes (as in 2018), considering that stress/strain from consecutive earthquakes may generate a stronger permeability increase following the second earthquake^54^, and iii) on a long-term scale, earthquakes increasing permeability may be more numerous than earthquakes decreasing permeability, with possible azimuthal^27^ and compression/extension effects dependences.

The main drivers controlling volcanic island hydrogeological system are classically lithology, primary permeability and weathering processes. On basaltic or hot spot island a decrease of permeability with geological age is generally observed^55,56^, but the recurrence of significant energy earthquakes is rather low in such geological context compared to subduction zones. We demonstrate here that this aging effect is not foremost on volcanic island in subductions zones. On the contrary, earthquake fracturing and associated unclogging mechanisms have a predominant effect inducing permeability increase, with faster effect than weathering processes. We can also assume that weathering processes and earthquakes act together to enhance aquifer permeability, the first altering the rock and the second cleaning the pathway and creating new fractures. Our results then confirm the hypothesis^57,58^ that the aquifer permeability increases with geological age on the andesitic type volcanic island, such as the one of Martinique, is the consequence of earthquakes seismic waves. In addition, we demonstrate the cumulative effect of extreme rainfall, to our knowledge the first such observations in earthquake hydrogeology.

**Figure 7:**
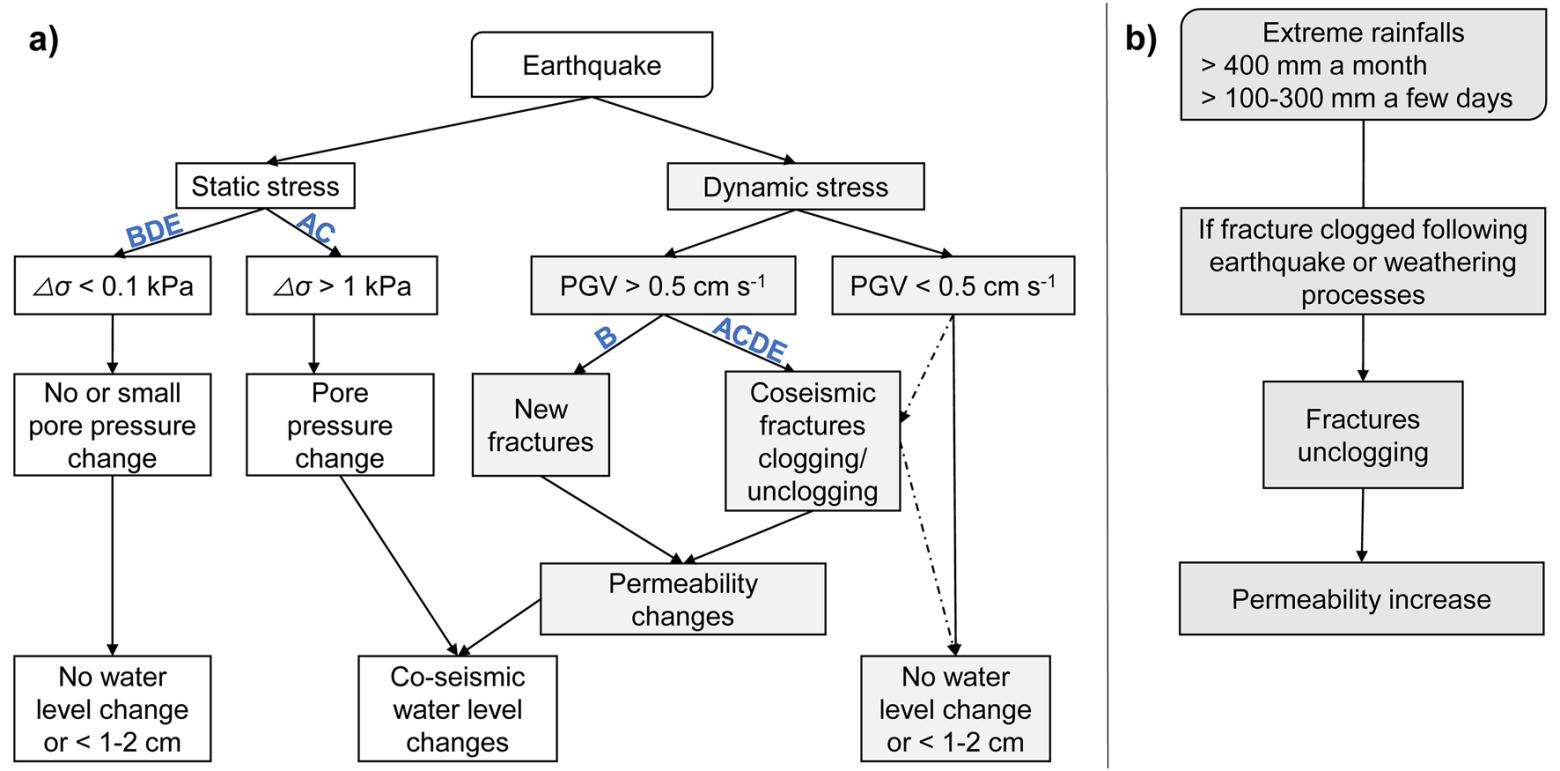
Schematic illustration of hydrogeological responses (**a**) to static and dynamic stress induced by earthquakes (A: 2007/11/29 earthquake; B: 2014/02/18; C: 2017/02/03; D: 2018/08/21; E: 2018/09/28) and (**b**) to extreme rainfalls events.

Figure 7 synthetizes the mechanisms occurring in the aquifer after earthquakes or extreme rainfalls. An impact of static stress change on the water level is possible for 2007 and 2014 earthquakes (denoted A and C on Fig. 7), but a change in static stresses cannot explain all the measured water level drop. However, we clearly observe (SI Appendix, Fig. S2) that dynamic stress are related to the observed co-seismic water level drop, for PGV higher than 0.5 cm s^−1^. Few mechanisms induced by dynamic stress can be put forward to explain increase or decrease of aquifer permeability in the Martinique island: clogging/unclogging of fracture due to remobilization of deposits and particles^2,8,9,15,16^, or creation of new fractures^10,17,18^. Fractures clogging in the next two months (earth tides analysis being calculated with 31 days span) following 2007 and 2017 earthquakes are transient as the permeability come back to its initial value in few years (Fig. 3). Fracture unclogging is the most realistic mechanism to explain the transient behavior of the permeability: the water pressure increase induced by the exceptional rainfall events (Fig. 6) should act as piston strokes strong enough to unclog congested fractures by colloids, particles or precipitates, as observed in karst flood^59^. Regarding the two earthquakes increasing permeability (2014/02/18 and 2018/09/28), two hypotheses are considered. Fracture unclogging is most likely concerning the 2018/09/28 earthquake as the phase shift (From 21° to 25°) and permeability (from 1.8 × 10^–4^ m^2^ s^−1^ to 2.4 × 10^–4^ m^2^ s^−1^) increase are relatively small compared to pre-earthquake value. Conversely, 2014 earthquake triggered significant phase shift (+ 5° to + 26°) and permeability increase (3.6 × 10^–5^ m^2^ s^−1^ to 2.5 × 10^–4^ m^2^ s^−1^), enabling to propose creation of new cracks/fractures as the main mechanism. The phase shift and transmissivity stability during the period 2014–2017, with no return to pre-earthquake values^9^, strengthens this hypothesis.

Nevertheless, quantifying relationship between the intensity of ground motion and hydrological or hydrogeological responses remains partly unclear. On one hand, a clear correlation between peak ground velocity (PGV) and a permeability change is highlighted in California^9^, but in another hand this correlation was not observed by others studies^27,60–62^. Threshold effects have also to be considered, as permeability changes should occur mainly above a threshold value^63^. The comparison of our results with those published in the literature (SI Appendix, Fig. S3) shows that our orders of magnitude are consistent with similar permeability variations for similar PGV observed in other contexts^9,27,59–61^. Some authors suggest a dependence on frequency of the oscillations^2^, but which frequency is more favorable is not demonstrated and neither do we see a clear frequency dependence on our data (SI Appendix, Fig. S4). An azimuthal dependence of the permeability changes has recently been suggested^27^. This hypothesis is interesting especially for the 2014/02/18 earthquake as this earthquake, inducing significant permeability increase (× 6), may open fractures, as the epicenter is situated due west with a regional scale fracture network SW-NE and NW–SE^34,64^. Nonetheless, it requires a detailed knowledge of the in-situ aquifer fracture distribution network to go further which in most cases is not known.

## Conclusion

In this study, we develop a new analytical model based on earth tides variations analysis to highlight permeability variations of a hydrogeological system induced by earthquakes dynamic stress. We also highlight unprecedented observations of the effect of heavy tropical rain and hurricanes on the permeability increase through fractures unclogging. Aquifer permeability increase with age is demonstrated over a decade and over 15 Ma. This finding has significant implications for groundwater flows within volcanic island and strengthens the importance of long-term water levels monitoring in wells, as properties of aquifers are changing with time.

Finally, our results should also be of interest for groundwater management, as a permeability increase on decade scales could rapidly affect water supply boreholes productivity, but also aquifer vulnerability (pesticides transfer process in the aquifer and towards the coastal zone, seawater intrusion, …). Aquitard permeability increase is also worrying as it decreases their ability to retain contaminants, for example under waste dumps, or under agricultural areas where pesticides are spread, thus weakening quality of groundwater resource. Permeability increase induced by intense rainfall could also be of interest as possible mechanism triggering landslides. Lastly, implications on groundwater flows within geothermal fields and actives volcanoes of the Lesser Antilles Islands are also intriguing perspectives, as geothermal field permeability enhancement could have implication on pressure equilibria. Furthermore, aquitard permeability enhancement of hydrothermally weathered volcanoes domes might influence eruptive reactivation processes.

## Methods

### Static and dynamic stresses

We use Okada model for a point source^48^ to calculate the static stress change and then evaluate the pore pressure change (assuming undrained conditions ∆p/B = ∆σ_kk_/3) at 50 m below the surface induced by the earthquakes. We use the focal mechanisms estimated from each earthquake. For some events (5 out of 8), we do not know the fault plane so we do the calculation for both nodal planes. The calculation is an estimation of the magnitude of the pore pressure variations for several reasons: (i) we do not know all fault planes so the sign of the variation cannot be determined, (ii) the assumption of point-source is not so true for magnitude 7 events, and (iii) there are large uncertainties in location (and even in focal mechanism in some cases) that should have a non-negligible impact on our calculation.

For example, three main localizations of the 2007 earthquake have been proposed with 20 km difference in the axis SW-NE and 25 km in depth between different agencies (OVSM, USGS and Geoscope). Depending on the location, the static stress variation ranges from 490 (Geoscope) to 9964 Pa (USGS) in absolute value, with water level change ranging from 3–4 cm (Geoscope) to 61–91 cm (USGS). Figure 1 show the localization by the OVSM-IPGP observatory of this earthquake, with the mechanisms calculated by Winter et al.^66^.

We use the seismic station located at the OVSM-IPGP observatory (14,735°/− 61,146°) to calculate dynamic stress, because it is the only station in Martinique that registered all the considered earthquakes (data available at https://volobsis.ipgp.fr/data.php). Maximum dynamic stress is estimated from PGV as G.PGV/V, assuming plane waves.

### Co-seismic water level drawdown

A 1D simple model^44^ that describes the post-seismic change in the hydraulic head at a given position x_B_ can be written as:

1 $$ h\left( {x_{B} ,t} \right) = h_{o} \mathop \sum \limits_{r = 1}^{\infty } \frac{{\left( { - 1} \right)^{r + 1} }}{{\left( {2r - 1} \right)}}\cos \frac{{\left( {2r - 1} \right)\pi x_{B} }}{2L}\exp \left( { - \left( {2r - 1} \right)^{2} \frac{t}{\tau }} \right) $$

This model^44^ consider a confined aquifer extending from x = 0 (at a local groundwater divide) and x = L (at a local discharge or recharge area). h_o _is the change in hydraulic head induced by the earthquake, i.e. h_o_ is a Dirac-source term (or sink term) homogenously distributed in all the aquifer. After the earthquake, the Dirac-source term diffuses through the aquifer with a characteristic time $\tau =\frac{4{L}^{2}}{\pi {D}^{2}},$ where D is the hydraulic diffusivity (D = T/S). The adimensional parameter  $\frac{{x}_{B}}{L}$ is known (ratio between the position of the borehole, and length of the aquifer) and $\frac{{x}_{B}}{L}\approx 0.84$. Then, the model is fitting at best to our data on Fig. 4, using two unknown parameters: h_o_ and $\tau$. Finally, the diffusivity is deduced from $\tau$, using a length L of 1400 m. The fitting has been done during the 48th first hours and the 10th first days (Fig. 4).

### Tidal response of a leaky aquifer

We first use Baytap08 software^40^, a modified version of Baytap-G^67^, to calculate the phase shift and amplitude ratio of the water-level response in the Galion borehole to M2 tidal component of earth tides. Water level data were divided into 31 days spans with an overlap of 15 days. Input data are presented in Fig. 2: hourly water levels, hourly barometric pressure at the nearest meteorological station (10 km NE), and hourly ocean tide fluctuations at the Fort-de-France harbor, re-projected to the Atlantic coastline of the island. The frequency analysis of each dataset is available in SI appendix Fig. S5.

The tidal response of a leaky confined aquifer model^43^ consists of an aquifer with a transmissivity *T* confined above by a semi-confining aquitard with a permeability, *k *and a thickness, *b. *The phase shift is defined as:

2 $$\eta =\mathrm{arg}\left[\frac{iwS}{\left(iwS+\frac{k}{b}\right)\xi }\right],$$

where arg(z) is the argument of the complex number* z*; *S* is storativity of the aquifer; *w* is the angular frequency related to the period of the tidal oscillation $\tau$ as $w = 2\pi /\tau$. The parameter ξ is defined as:

3 $$\xi =1+{\left(\frac{{r}_{c}}{{r}_{w}}\right)}^{2}\frac{i w {r}_{w }{K}_{0}(\beta {r}_{w})}{2T\beta {K}_{1}(\beta {r}_{w})},$$

where ${r}_{c}$ is the well casing (${r}_{c}$ = 0.056 m) and ${r}_{w}$ is the radius of the screened portion of the well (${r}_{w}$ = 0.076 m); ${K}_{0}$ and ${K}_{1}$ are the Bessel function of the second kind of the zero and first order, respectively. The parameter β is given as:

4 $$\beta ={\left(\frac{k}{Tb}+\frac{iwS}{T}\right)}^{1/2},$$

To summarize, in the tidal response of a leaky confined aquifer model^43^ the phase shift $\eta$ for a given tidal oscillation depends on three unknown parameters: the storativity *S* and transmissivity *T* of the aquifer and the ratio permeability versus thickness of the aquitard *k/b.* These three parameters may nevertheless change in time,* t*. The modeling of co-seismic water level drawdown (Fig. 4) highlights low variability over the last 13 years, thus it is a fair approximation to assume that T/S is constant over time and from our modeling of co-seismic water level we can fix T/S = 3.

Earthquakes modify the stress field and we assume that the permeability of the aquifer and the aquitard varies exponentially with stress^45,46,68^. This last approximation will give a relationship between the T and k/b. Indeed, one can write:

5 $$ T\left( t \right) = T_{0} e^{ - \gamma \vartriangle \sigma \left( t \right)} \;{\text{and}}\;\frac{k\left( t \right)}{b} = \frac{{k_{0} }}{b} e^{{ - \gamma^{\prime } \vartriangle \sigma \left( t \right)}} , $$

where ${T}_{0}$ and ${k}_{0}$ are respectively the transmissivity of the aquifer and permeability of the aquitard for a reference state. $\vartriangle \sigma (t)$ is the change in effective stress between the reference state and the state at a time* t*. $\gamma$ and $\gamma^{\prime }$ are properties of the aquifer and the aquitard. Equation (5), leads to a relationship between T and k and:

6 $$ \frac{k\left( t \right)}{b} = \frac{{k_{0} }}{b}\left( {\frac{T\left( t \right)}{{T_{0} }}} \right)^{{\frac{{\gamma^{\prime } { }}}{{\gamma { }}}}} . $$

For the reference state, one can choose 2019, as a pumping test was done and gave a transmissivity for the aquifer *T*_0_ = 2.6 × 10^−4^ m^2^ s^−1^. During the weeks before the pumping test, the phase shift value related to M2 earth tide wave was 25.5°, which leads using Eq. (2) with the assumption that T/S = 3 to $\frac{{k}_{0}}{b}=6.3 \times {10}^{-9}$ s^−1^ . Phase shift values of M2 earth tide waves are available from 2006 to 2019. However, we only have two shift values of O1 earth tide waves one in 2008 and one in 2017. Using the O1 phase shift of 1° in 2008 and the associated M2 phase shift (− 8°), one gets from Eq. (2) $T(2008)=8.7 \times  {10}^{-6}{{{\text{m}}}^{2} s}^{-1}$ and $\frac{k}{b}(2008)=2.2 \times  {10}^{-11}$ s^−1^. Finally, using the results obtained in 2008 and 2019 as the reference state, one gets from Eq. (5): $\frac{{\gamma ^{\prime } }}{{\gamma}} = 1.6$.

Using Eq. (6)—with the assumption that T/S remains constant—implies that the phase shift $\eta$ for a given tidal oscillation depends only on one parameter: the transmissivity of the aquifer or the permeability of the aquitard, permeability of the aquitard and transmissivity of the aquifer being related by Eq. (6). The results using the phase shift value M2 earth tide is given on Fig. 3. The model was also tested for the phase shift of O1 earth tide available in 2017, the model predicts a value of 45°, a value very closed to the one observed (44°).

Note that we only focus on the phase shift and not on the amplitude ratio. Indeed, the amplitude ratio requires knowledge of two poroelastic parameters, which may also change in time^43^.

In order to evaluate the influence of the assumption that T/S remains constant, we test our model considering S = 10^–4^, a hypothesis classically used by authors using Hsieh model^6^. Results, shown in SI appendix, Fig. S6, are close to the ones of Fig. 3. Choosing a T/S constant or a storativity constant is not predominant in the calculations. Comparison is presented in SI appendix, Fig. S7.

## Supplementary information

Supplementary Information.

## Data Availability

Daily water level monitoring data (Fig. 2a) comes from the BRGM piezometric monitoring network of Martinique, set up since 2005 as part of the European Water Framework Directive and is funded by the French Ministry of Environment and BRGM. Data are available online: https://ades.eaufrance.fr/. The borehole national reference is BSS002NNZL (1175ZZ0154/NF4). Two sensors have used both with millimetric resolution. OTT Thalimedes from 2005 to 2014, a float operated shaft encoder with integrated data logger, and SEBA Dipper-PT data logger, from 2014 to 2019, a ceramic pressure sensor, with integrated air pressure compensation tube. Public reports of felt earthquakes in Martinique by the OVSM-IPGP observatory are available online: https://volcano.ipgp.fr/martinique/Communiques.htm. Historical earthquakes database is available online: https://sisfrance.net (temporarily hosted at https://sisfrance.irsn.fr/). Atmospheric data (Figs. 2b and 6a) have been purchased from the French meteorological agency (Météo-France) and are subject to a disclaimer of diffusion. Hourly ocean tide data of the Fort-de-France harbor (Fig. 2c) are available online: https://dx.doi.org/10.17183/REFMAR#126. Earthquake focal mechanisms are from USGS (https://earthquake.usgs.gov) (2007/11/29, 2008/02/06, 2010/01/12, 2014/02/18, 2015/07/16 and 2018/08/21), from Geoscope (https://geoscope.ipgp.fr/index.php/en/data/earthquake-data/catalogs-of-earthquakes) (2007/11/29), from Corbeau et al.^65^ for 2017/02/03 and 2018/09/28 earthquakes and from Winter et al.^66^ for 2007/11/29 earthquake.
