# Supplementary material for: Earthquakes and extreme rainfall induce long term permeability enhancement of volcanic island hydrogeological systems
Source: Sci Rep. 2020 Nov 19;10:20231. doi: 10.1038/s41598-020-76954-x (PMC7677319; doi:10.1038/s41598-020-76954-x)
Supplement: Supplementary file 1 — Supplementary Information. [file 41598_2020_76954_MOESM1_ESM.pdf]

Table S1 : Static and dynamic stress calculations for the five more important earthquakes over the period 2007-2019 (Seismic energy higher than 1.6 10^{-2} $J.m^{-3}$) triggering co-seismic water level drop (white lines). Light gray lines show calculations for three other earthquakes, with seismic energy close to 10^{-2} $J.m^{-3}$; but these earthquakes did not trigger water level drop. Finally, the last calculation concerns a significant teleseism, the 2010/01/12 M7 Haiti earthquake at an epicentral distance of 1300 km (dark grey line), also with no water level impact.

Date	UT time	Lat.	Long.	Mag.	Depth (km)	Epicentral distance (km)	Hypocentral distance (km)	Seismic energy density ($J.m^{-3}$)	$\|\Delta p\|/B$ (kPa)	Δz (B=0.9) (cm)	Δz (B=0.6) (cm)	PGV ($cm.s^{-1}$)
2007/11/29	19:00	14.99	-61.03	7.4	160	38	152	8.10E-01	1.6	15	10	2.8
2008/02/06	18:37	15.05	-60.20	5.3	19	78	80	4.92E-03	0.03	0	0	0.7
2009/11/28	04:18	14.72	-60.81	4.0	9	16	18	5.87E-03	-	0	0	0.01
2010/01/12	21:53	18.44	-72.57	7.0	13	1300	1300	3.19E-04	0.007	0	0	0.1
2014/02/18	09:27	14.72	-59.07	6.5	15	203	203	1.65E-02	0.29	3	2	0.6
2015/07/16	15:16	13.87	-58.55	6.4	11	270	271	9.71E-03	0.09	1	1	0.4
2017/02/03	19:54	14.94	-60.39	5.6	38	64	74	1.73E-02	0.03	0	0	2.1
2018/08/21	21:31	10.74	-62.91	7.3	112	485	498	1.60E-02	0.1	1	1	0.5
2018/09/28	12:32	15.09	-60.36	5.4	49	76	90	3.62E-02	0.01	0	0	1.3

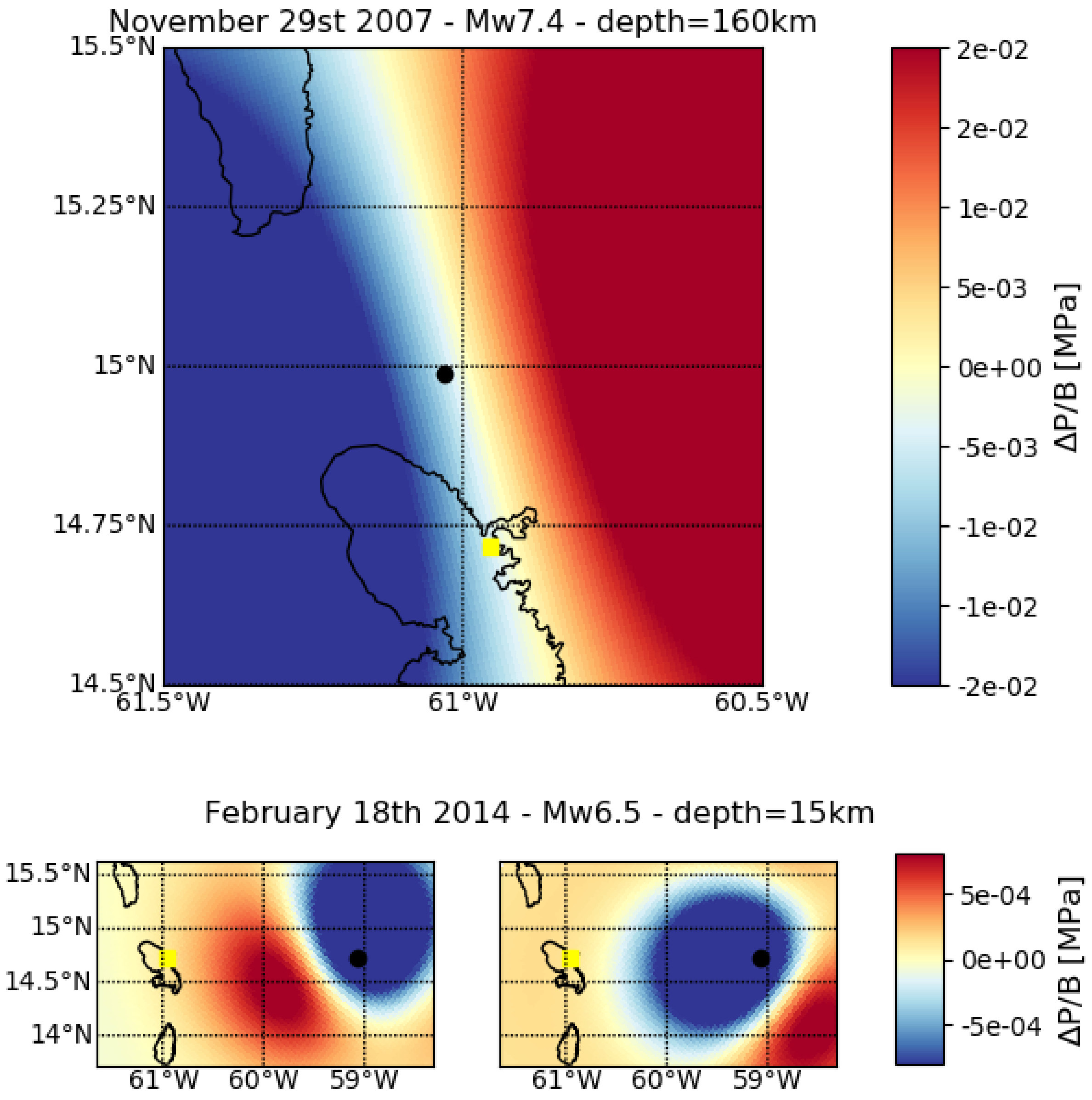

Figure S1: Static mean stress change ⊿σ calculated in an elastic half-space with the Okada Model [Okada, 1992] for the two earthquakes with the highest variations, at the Galion borehole, of 1.6 kPa (2007/11/29) and 0.3 kPa (2014/03/02). The black dot indicates the epicenter of the earthquakes and the yellow dot the location of the Galion borehole. Since we do not know which nodal plane is the fault plane for the 2014 earthquake, the bottom part shows the two possible solutions considering each nodal plane. The assumption made are presented in the method section. Assuming undrained conditions and a Skempton coefficient Between 0.6 and 0.9 [Roeloffs, 1996; Freed, 2005], the change in pore pressure induce a change in water level of ~10-15 cm for 2007/11/29 earthquake and 2-3 cm for the 2014/03/02 earthquake. For these two events, an impact of static stress change on the water level is clearly possible, as observed variations at the Galion piezometer are 43 cm and 8 cm, respectively (Fig. 2a and 2d). However, static effect changes are not able to explain all groundwater changes observed.

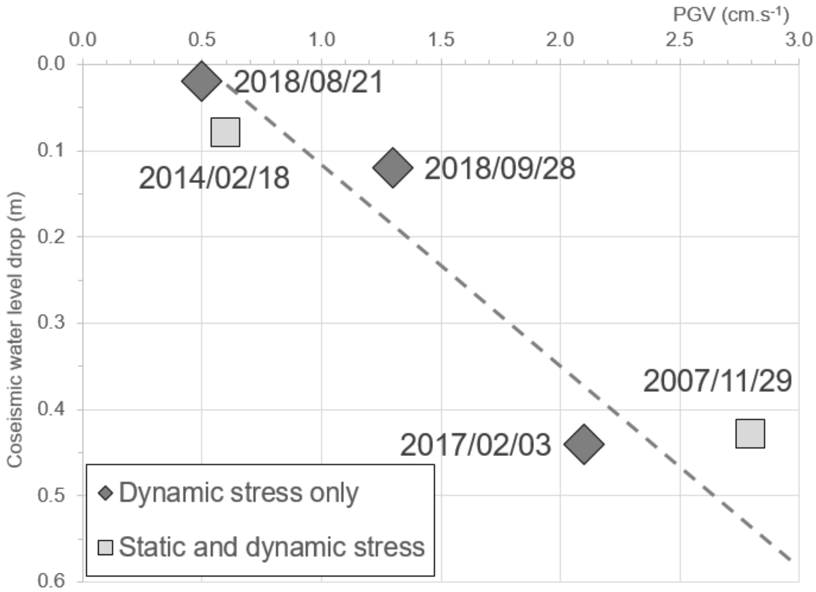

Figure S2 : Co-seismic water level drop as a function of Peak Ground Velocity (PGV). Higher water level drop is related to higher PGV.

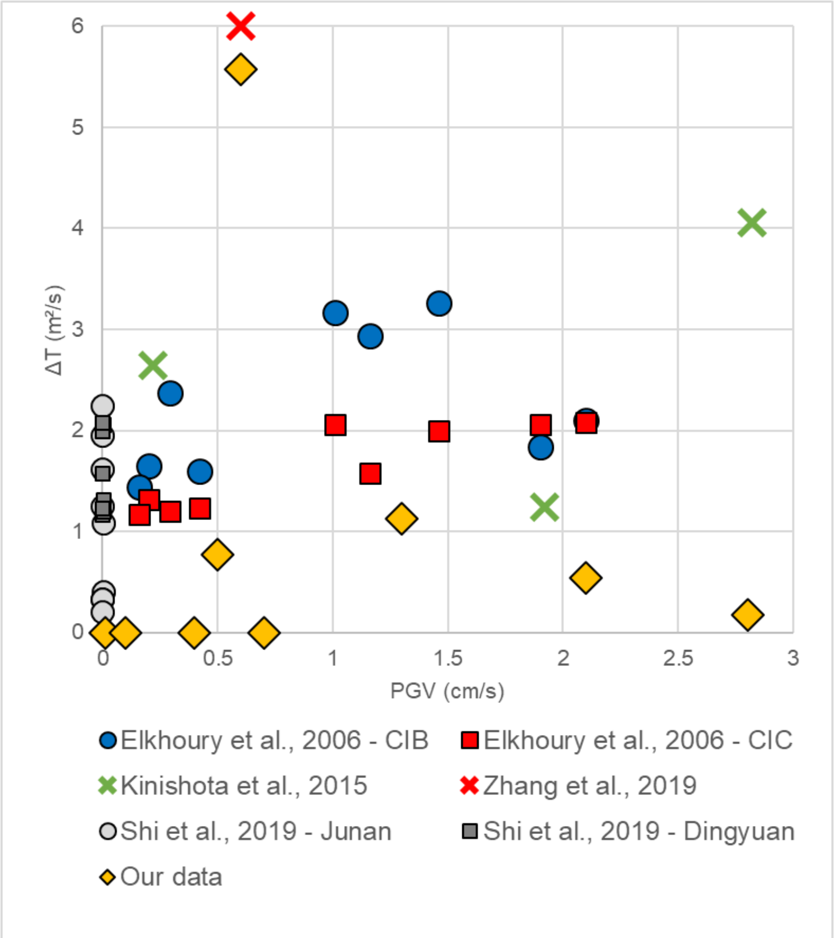

Fig. S3: Transmissivity change before and after each earthquake plotted against peak ground velocity (PGV, in cm.s^{-1}) calculated at the OVSM seismic station: comparison between our results and those published in the literature.

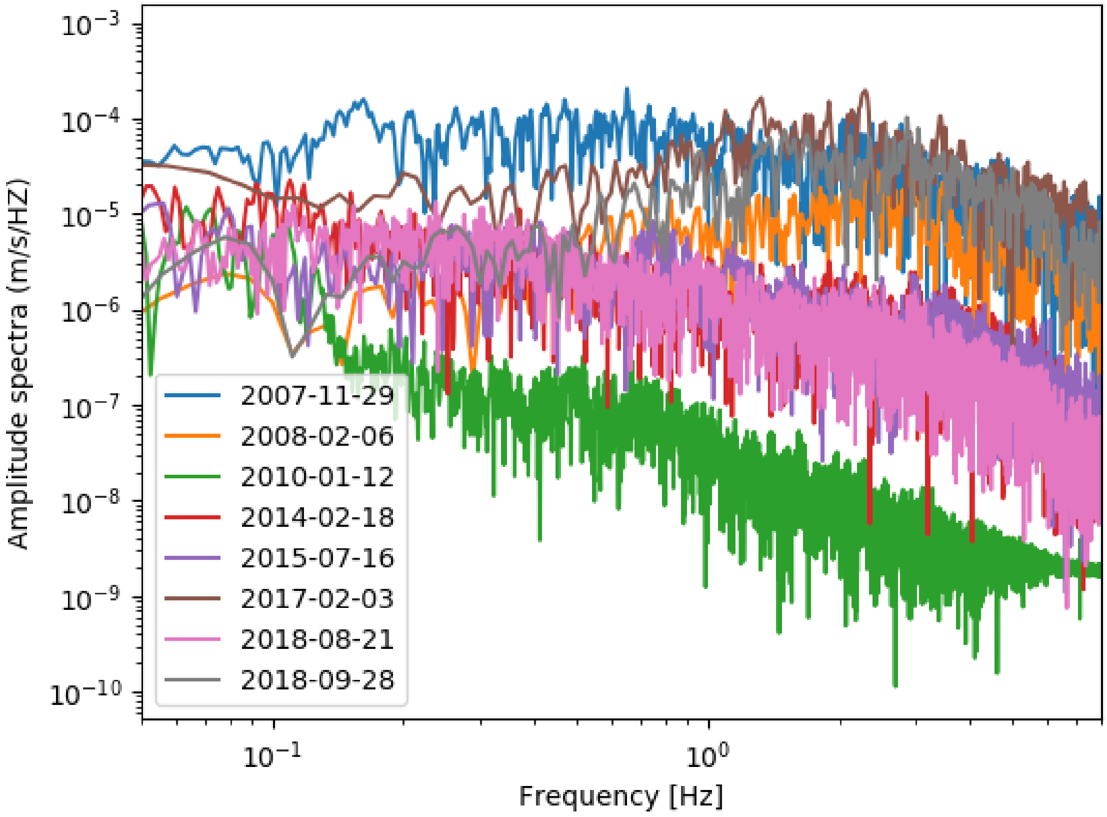

Figure S4: Frequency analysis of seismic wave vertical velocity at the OVSM seismic station. Manga et al. [2012] and references therein suggest a dependence on frequency of the oscillations, that could explain why the maximum dynamic shear stress is low while the effect of the earthquake is high, but we do not see a clear dependence on our data.

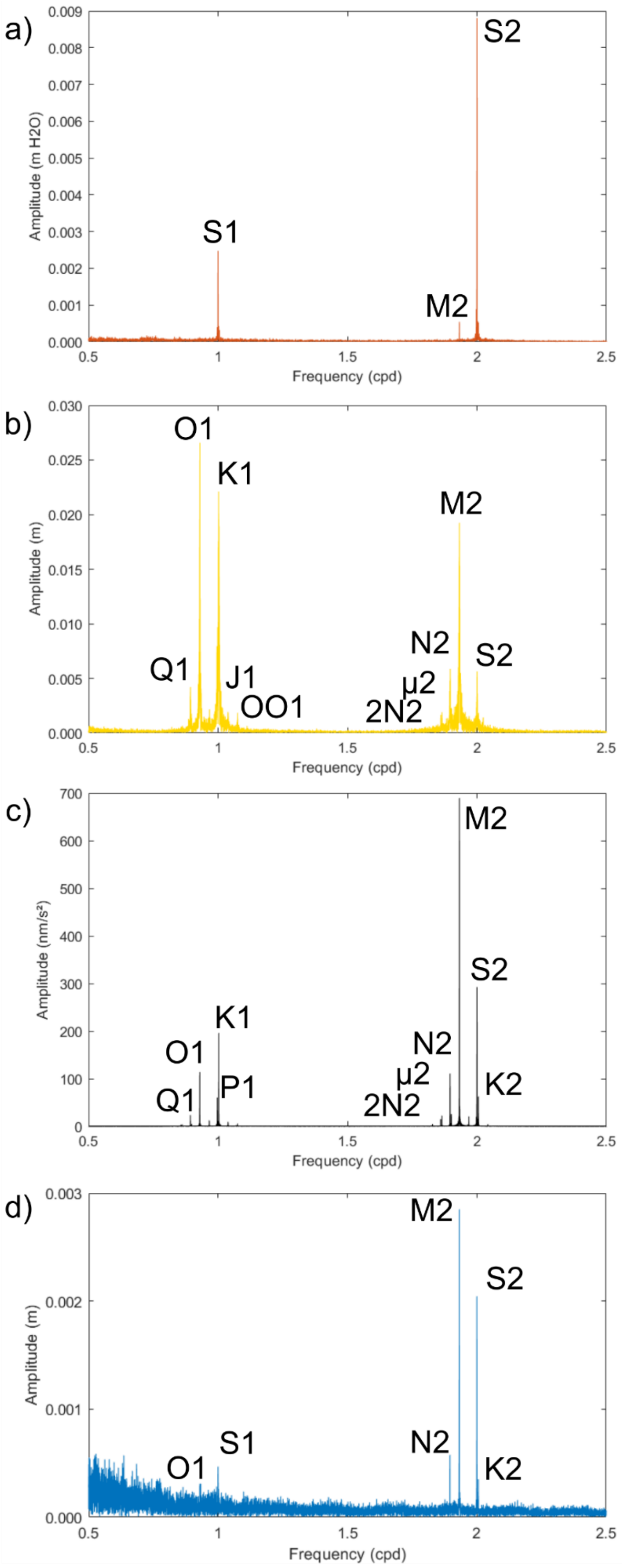

Fig. S5: Spectral analysis: results of Fast Fourrier Transform (FFT), with hourly data from 2006 to 2019, showing the different tidal harmonic of a) barometric pressure, b) tide gauge station (Fort-de-France harbor tide gauge), c) Earth-tide at the Galion borehole calculated by T-Soft and d) water-level in the Galion borehole. cpd: cycle per day.

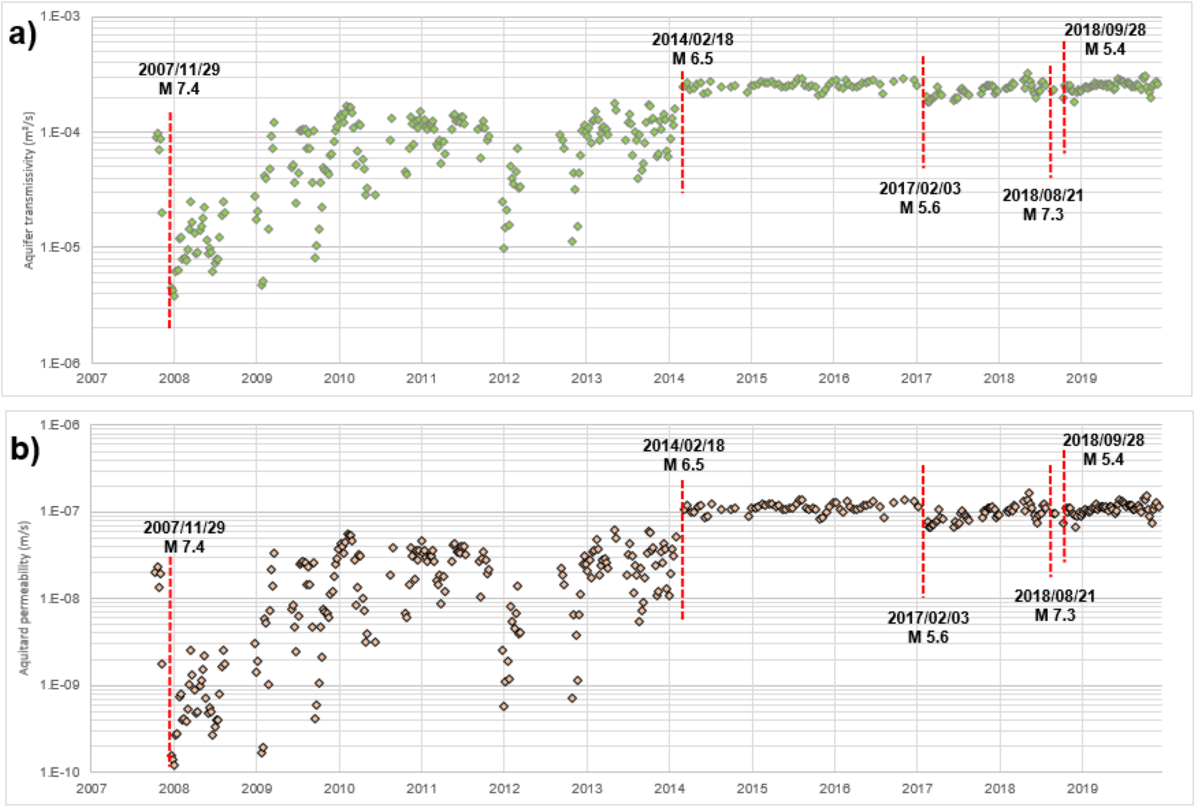

Figure S6: a) Aquifer transmissivity and b) aquitard permeability evolutions over 12 years considering $S=10^{-4}$

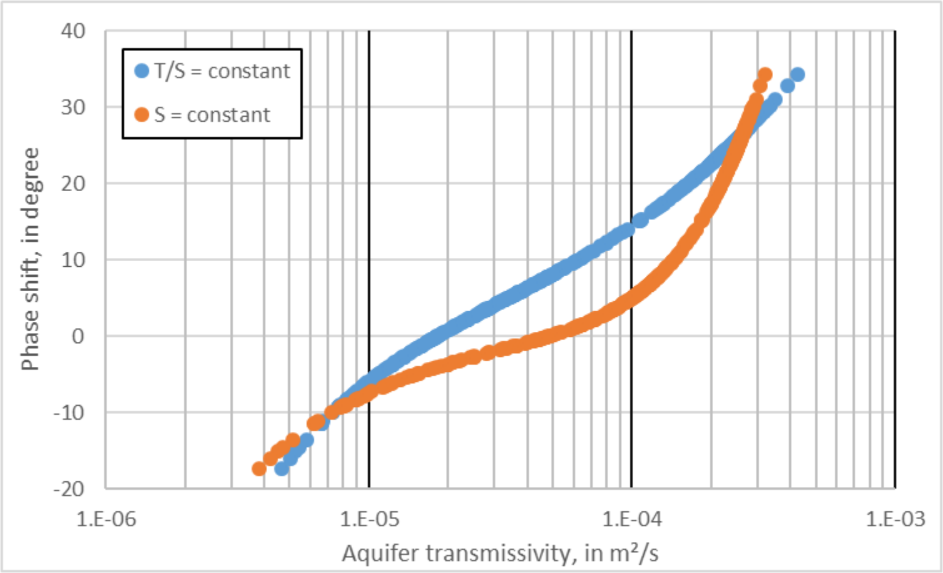

Figure S7: Relationship between M2 phase shift and aquifer transmissivity following the two assumptions: T/S= constant (in bleu) or S=constant (in orange).
